# Effect of Steel Hardness and Composition on the Boundary Lubricating Behavior of Low-Viscosity PAO Formulated with Dodecanoic Acid and Ionic Liquid Additives

**DOI:** 10.1021/acs.langmuir.1c02848

**Published:** 2022-02-23

**Authors:** Wahyu Wijanarko, Hamid Khanmohammadi, Nuria Espallargas

**Affiliations:** † Norwegian Tribology Center, Department of Mechanical and Industrial Engineering, Norwegian University of Science and Technology (NTNU), Trondheim 7491, Norway; ‡ Department of Mechanical Engineering, Sepuluh Nopember Institute of Technology (ITS), Surabaya 60111, Indonesia

## Abstract

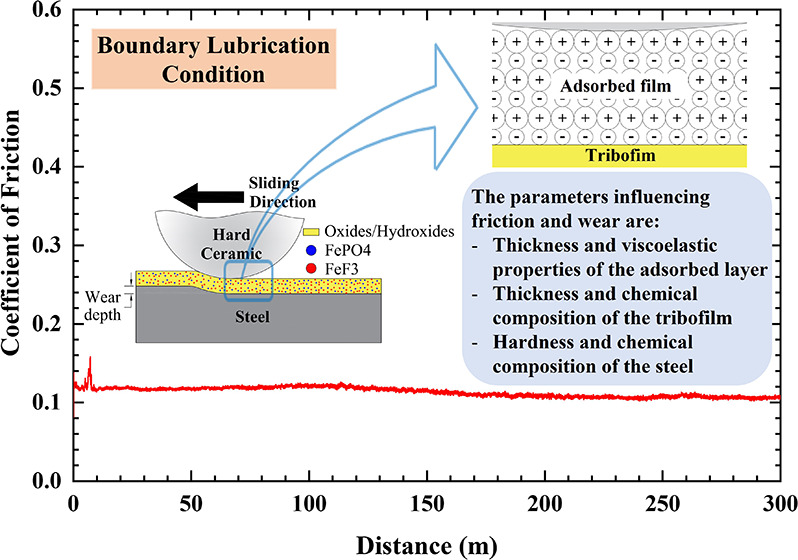

Two ionic liquids, tributylmethylphosphonium dimethylphosphate (PP) and 1-butyl-1-methylpyrrolidinium tris(pentafluoroethyl)trifluorophosphate (BMP), as lubricant additives in polyalphaolefin (PAO8) were studied under boundary lubricating conditions on two types of steel (AISI 52100 bearing steel and AISI 316L stainless steel). The tribological behavior of these ILs was compared with dodecanoic acid, a well-known organic friction modifier. This study employs a ball-on-disk tribometer with an alumina ball as a counterpart. A range of advanced analytical tools are used to analyze the tribofilms, including scanning electron microscopy equipped with a focused ion beam, scanning transmission electron microscopy equipped with X-ray energy-dispersive spectroscopy, and X-ray photoelectron spectroscopy. A quartz crystal microbalance with dissipation was used to study the surface adsorption of the additives on iron- and stainless steel-coated sensors to reveal the adsorption kinetics, adsorbed layer mass, and bonding strength of the adsorbed layer on the metallic surfaces. The most important factors controlling friction and wear are the thickness and viscoelastic properties of the adsorbed layer, the thickness and chemical composition of the tribofilm, and the hardness and chemical composition of steel. Among all additives studied, BMP on stainless steel gives a strongly adsorbed layer and a durable tribofilm, resulting in low friction and excellent antiwear properties.

## Introduction

Friction between moving parts and their associated wear is estimated to be directly responsible for 23% of the world’s energy consumption.^1^ Road transport is responsible for 22% of Europe’s CO_2_ emissions. An electric car charging on the European electricity grid corresponds to about 20 g/km of CO_2_ emissions. In electric cars, moving parts work at a higher speed than in internal combustion engine cars, making the lubricants function more as a torque transfer than as a load-bearing.^2^ The higher the speed of the tribological component, the higher the temperature generated in the lubricant. Therefore, low-viscosity lubricants with better cooling properties and higher temperature stability are the trend for meeting the UN sustainable goals.^3,4^ The performance of low-viscosity lubricants can be maintained by advancing the technology of additives with multiple functions, for example, simultaneous friction-reducing and antiwear properties. In recent years, researchers have attracted great interest in ionic liquids (ILs) since they are seen as potential high-performance lubricant additives due to their inherent polarity, which provides strong surface adsorption. Moreover, ILs can be easily tailored and tuned to meet different properties; therefore, they are potential candidates for multifunctional lubricant additives.

ILs are organic salts with a low melting point (below 100 °C).^5,6^ ILs consist of cations and anions with an asymmetric structure and delocalized electrical charges, preventing them from forming solid crystals. As a result, ILs are liquid at room temperature. ILs were first studied as an alternative to space lubricants in the early 2000s due to their unique properties, such as nonflammability, low melting point, low volatility, high thermal stability, and high polarity.^7−10^ Since then, the tribological performance of ionic liquids has been compared with conventional hydrocarbon-based lubricants, such as perfluoropolyether (PFPE),^11−13^ polyalphaolefin (PAO),^13,14^ and mineral oils.^14−16^ However, due to their complex synthesis and price, recent works on ILs have focused on their performance as lubricant additives.^17−22^

Two lubrication mechanisms of ILs are proposed in the literature: (1) ILs adsorb to the worn surface to form adsorbed layers and (2) ILs react with the worn surface to form a protective tribofilm.^19^ For the first mechanism, the rubbing action during the test removes the electrons from the metal surface, leaving a positively charged surface.^23^ The anion moieties of ILs are attracted to the surface, while the cation moieties face the lubricant, forming the first adsorbed layer. Subsequently, the adjacent IL in the lubricant is attracted to the first adsorbed layer in the same manner, forming a multilayer structure at the surface.^24−27^ For the second mechanism, localized high temperature and high pressure are generated at the contact area, decomposing the ILs. Consequently, the decomposition products of ILs reacts with the nascent worn surface to form a protective tribofilm.^28−32^ Most research on ILs as lubricant additives focuses on the boundary lubricating condition and tribofilm formation.^28−38^ Only a few study the adsorption mechanisms of ILs;^22,35,39^ therefore, the lubricating mechanism of ILs is still far from being fully understood.

This paper studies ILs as potential additives in a low-viscosity nonpolar medium. Two ILs (tributylmethylphosphonium dimethylphosphate and 1-butyl-1-methylpyrrolidinium tris(pentafluoroethyl)trifluorophosphate) have been studied in a polyalphaolefin base lubricant with a viscosity of 8 cSt at 100 °C (PAO8). No additional additives were used to isolate the effect of the ILs alone. The lubricating mechanisms of ILs are compared with a well-known organic friction modifier (dodecanoic acid). The lubricating mechanisms have been investigated by studying their behavior on two steel materials (AISI 52100 bearing steel and AISI 316L austenitic stainless steel) due to their wide range of applications in tribological components. Therefore, the effect of surface chemistry and mechanical properties on the lubricating mechanisms could be investigated. For the surface adsorption study, a quartz crystal microbalance with dissipation mode (QCM-D) using iron- and stainless steel-coated sensors was employed. X-ray photoelectron spectroscopy (XPS) and scanning transmission electron microscopy equipped with X-ray energy-dispersive spectroscopy (STEM-EDS) were used to study the tribofilm formation.

## Materials and Methods

### Materials

Two ionic liquids (ILs) were used as additives in a nonpolar lubricant, i.e., tributylmethylphosphonium dimethylphosphate (abbreviated as PP) and 1-butyl-1-methylpyrrolidinium tris(pentafluoroethyl)trifluorophosphate (abbreviated as BMP). A well-known organic friction modifier is used as a reference, i.e., dodecanoic acid (abbreviated as C12). Polyalphaolefin with a viscosity of 8 cSt at 100 °C (abbreviated as PAO) was chosen as the base lubricant. PP (97% purity and a molar mass of 342.40 g/mol) was purchased from Fluorochem. Both BMP (≥98%, 587.27 g/mol) and C12 (≥99%, 200.32 g/mol) were purchased from Sigma-Aldrich. Meanwhile, PAO was obtained from Chevron Phillips Chemical. All chemicals were used as received without further purification. Table 1 shows the chemical formula, density, and chemical structure of all additives used in this study. The selected additive concentrations are 1 and 0.1 wt % for ILs and C12, respectively. The concentrations of ILs are chosen based on a two-way ANOVA analysis performed by us in an independent work. The optimum concentration for ILs in PAO was 1 wt % (different concentrations were tested: 0.25, 0.5, 1, and 2 wt %). In contrast, 0.1 wt % was the optimum concentration for C12.^40^ A magnetic stirrer was used to blend the base lubricant and the additive for 4 h at 70 °C, followed by 20 h at room temperature.

**Table 1:**
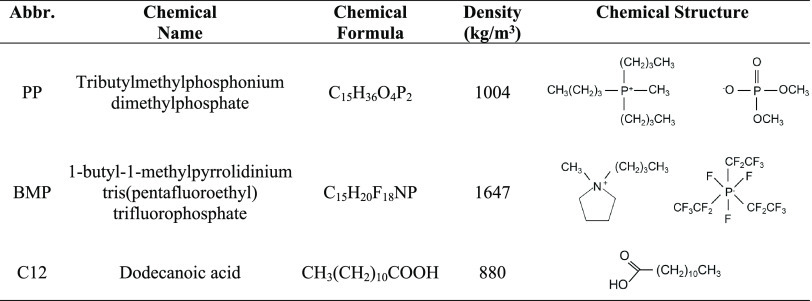
Chemical Formula, Density, and Chemical Structure of All Additives

The tribological performance of each lubricant was studied on AISI 52100 bearing steel and AISI 316L stainless steel. Both steels were purchased from Smith Stål (Trondheim, Norway) with hardness values of 60 HRC and 217 HB (equivalent to 18 HRC) for AISI 52100 bearing steel and AISI 316L stainless steel, respectively. The elastic modulus and Poisson’s ratio of both steels are 210 GPa and 0.29, respectively. Disk samples with a thickness of 6 mm were prepared from a 30 mm diameter rod. Surface preparation was done by following the procedure in the metalog guide provided by Struers for each material until it reached a surface finish of Ra = 0.090 ± 0.003 μm.^41^ After surface preparation, the sample disks were ultrasonically cleaned in a distilled water–ethanol mixture (ca. 1:1) for 5 min, then rinsed with fresh ethanol, and dried with pressurized air.

### Testing and Characterization Methods

The stability of the lubricant mixture at room temperature was examined by a turbidity meter (Hanna Instruments HI-88713). The lubricants were put in an ultrasonicator for 1 h before the test. The turbidity number (FNU) was measured by calculating the average value of 12 measurements taken from 2 h tests. PAO, PAO-C12, and PAO-PP have stable turbidity for 2 h, transparent and without phase separation. On the other hand, PAO-BMP resulted in the highest turbidity numbers of all mixtures, indicating lower solubility. However, the turbidity numbers were stable throughout the test. The dynamic viscosity of the lubricants was measured using a rheometer (Haake Mars Rotational Rheometer, with a CC27 cylinder measuring system, with the built-in Peltier element). The measurements were conducted by applying a shear rate of 500 s^–1^ for 30 s at 23 °C in humid air. The measured viscosity of the PAO base lubricant was 81 mPa·s. The addition of C12 did not change the viscosity of the base lubricant, and the ILs slightly increased the lubricant’s viscosity to 87 and 85 mPa·s for PAO-PP and PAO-BMP, respectively. The density of the lubricants was measured by the weighing method at constant volume. The measured density of the PAO base lubricant was 831.8 kg/m^3^. The addition of the additives slightly increased the density of the lubricant to 840.9, 853.6, and 840.8 kg/m^3^ for PAO-PP, PAO-BMP, and PAO-C12, respectively.

The tribological tests were performed using a unidirectional ball-on-disk tribometer (Anton Paar with Phoenix tribology software) to evaluate the tribological performance of each lubricant on AISI 5200 steel and AISI 316L stainless steel. The tests were conducted using a stationary alumina ball against a rotating disk sample of AISI 5200 or AISI 316L stainless steel under boundary lubricating conditions. The alumina ball (fused ceramic) was purchased from Precision Ball and Gauge Co., Ltd with an elastic modulus of 300 GPa and a Poisson’s ratio of 0.21. The roughness of the alumina ball was 0.025 μm. The test parameters were as follows: a ball diameter of 6 mm, a free-weight load of 20 N (corresponding to a maximum initial contact pressure of 1.96 GPa), a disk rotation speed of 40 rpm, and a rotation track diameter of 10 mm. From these parameters, the calculated lambda (λ) value according to the EHL Hamrock–Dowson equation is 0.039 for the PAO base lubricant (the pressure-viscosity coefficient of PAO is 13 GPa^–1^ at 25 °C);^42^ therefore, the boundary lubricating condition is met. The calculated λ value for PAO-PP, PAO-BMP, and PAO-C12 was 0.040 (assuming the same pressure-viscosity coefficient for additivated PAO as the PAO base lubricant), indicating that the lubricating regime was still in the boundary condition. All lubricants were tested for a distance of 300 m (4 h) at room temperature. For each lubricant–substrate combination, at least two tests were performed to verify the repeatability of the results.

The wear volume was quantified using an optical three-dimensional (3D) microscope (Alicona Infinite Focus Microscope, IFM), followed by surface image analysis using MountainsMap software. The wear volume was measured from four wind directions of the wear tracks, and the average value was then calculated. After that, the specific wear rate was calculated by the following equation^43^
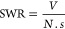
1where SWR is the specific wear rate (mm^3^/Nm), *V* is the volume loss (mm^3^), *N* is the normal load (*N*), and *s* is the sliding distance (*m*). The average SWR value and the standard deviation of each lubricant–substrate combination were reported.

The wear track top surface was observed using a Quanta FEG 650 scanning electron microscope (SEM). The wear track secondary electron images were recorded using an Everhart–Thornley detector (ETD). The wear track cross section was prepared and studied using an FEI Helios Nanolab DualBeam scanning electron microscope with a focused ion beam (SEM-FIB). A gallium liquid metal ion source was used for preparing the cross section by deposition, milling, and polishing processes. To protect the wear track surface from damage, double layers of platinum were deposited in sequence before milling and polishing processes. The secondary electron images of the cross section were taken using a through lens detector (TLD). After the images were recorded, the process was continued to make a thin lamella sample with a thickness of less than 60 nm using the same SEM-FIB. The tribofilm characterization and chemical composition were studied by examining the lamella by scanning transmission electron microscopy (STEM, Hitachi SU9000) equipped with an X-ray energy-dispersive spectroscopy (EDS) detector (Ultim Extreme, Oxford Instruments).

The elemental composition inside the wear track was examined by X-ray photoelectron spectroscopy (XPS, Kratos Axis Ultra DLD) with monochromatic Al Kα as the X-ray source with 10 kV accelerating voltage and 10 mA current. The sample analysis chamber was set to vacuum with a pressure of 9 × 10^–9^ Torr during the acquisition. Electrostatic and hybrid lenses were used for AISI 52100 steel and AISI 316L stainless steel samples. A high-resolution scan with 20 eV pass energy and 0.1 eV step size was selected to collect the elemental data of phosphorus (P) and fluorine (F). To study the tribofilm, depth profiling was done by sputtering the surface with Argon ions with the following parameters: a pressure of 4.4 × 10^–7^ Torr, an energy of 4 kV, and a raster size of 2.5 × 2.5 mm. The selected sputtering times were 5 and 85 s. A sputtering time of 5 s was used to remove the contamination on the surface, and a sputtering time of 85 s was used to study the tribofilm chemical composition. The XPS elemental data were analyzed by CasaXPS software using the curve-fitting parameters shown in Table 2 for detailed quantification.

**Table 2:** Detailed Curve-Fitting Parameters of Compounds Used for XPS Characterization

signal	binding energy (±0.1 eV)	FWHM (±0.1 eV)	line shape	assignment
F 1s^44,45^	684.9	1.6	GL(30)	F^–^
P 2p^46,47^	133.7	1.6	GL(30)	(PO_4_^3–^)

The adsorption studies were performed using a quartz crystal microbalance with dissipation (QCM-D, Biolin Scientific). Two sensors were used in this study, i.e., an iron (Fe)-coated sensor and a stainless steel (SS)-coated sensor from Biolin Scientific. Before the experiment, the sensors were cleaned based on the cleaning procedure provided by Biolin Scientific. The experiment was started by injecting the base lubricant into the sensor and monitoring the frequency and dissipation shift for at least 30 min to obtain a steady baseline. After that, the solution was changed to the formulated lubricant (base lubricant and additive) for 2 h to measure the adsorption behavior of the additive. Then, the solution was changed back to the base lubricant for 1 h to remove the weakly bond additive and measure the frequency and dissipation shift of the strongly adsorbed additive species. The experiment was performed with a flow rate of 50 μL/min using a peristaltic pump. Only the frequency and dissipation change of the fundamental frequency (1st overtone) could be recorded because the viscosity of the tested lubricants was high enough to dampen the quartz crystal, resulting in a high noise-to-peak ratio at higher overtones. At least two experiments were conducted for each solution to check the repeatability of the results.

## Results

### Tribological Testing

**Figure 1:**
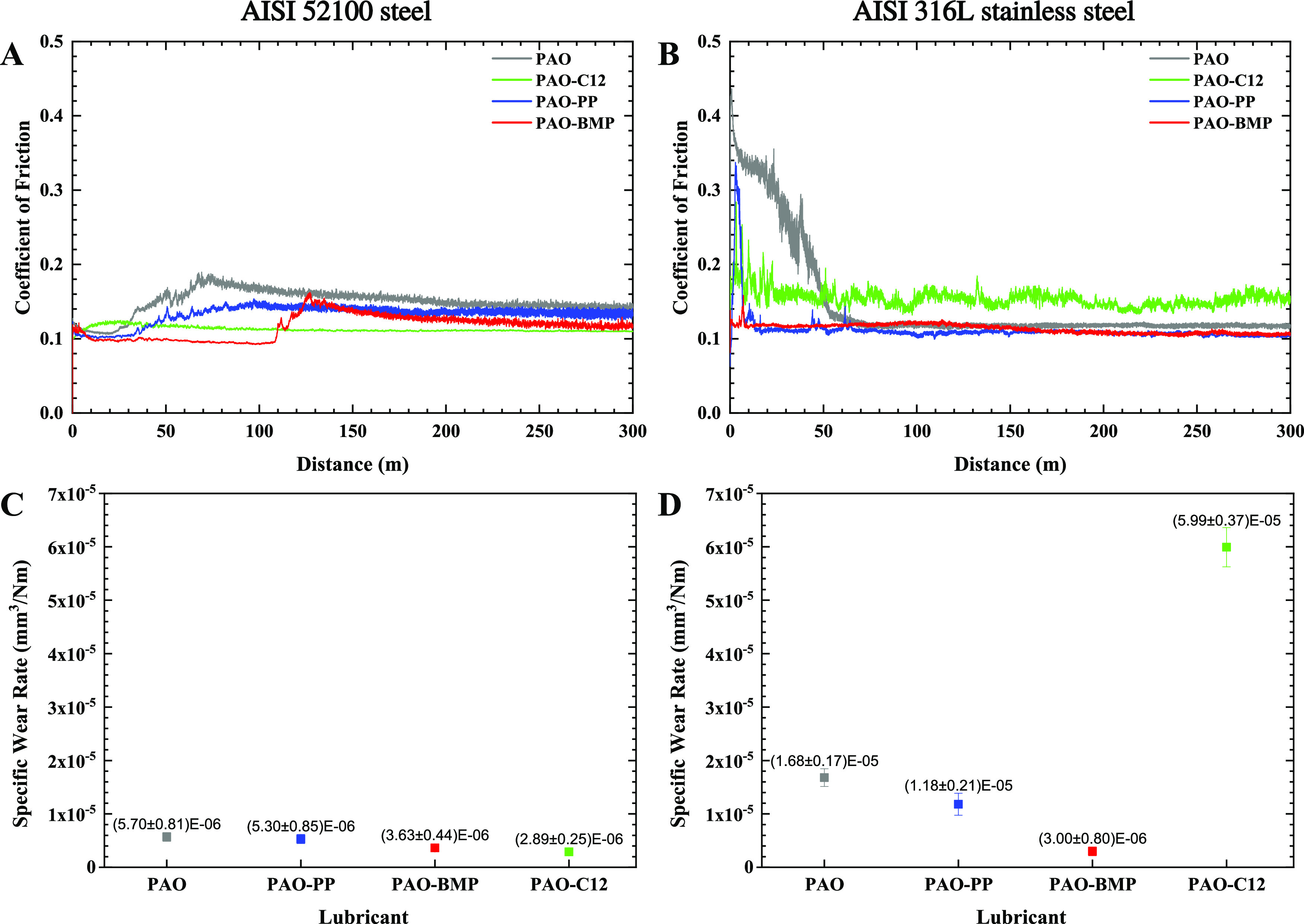
Friction evolution and specific wear rate of AISI 52100 steel (A, C) and AISI 316L stainless steel (B, D) lubricated by PAO with and without additives.

The effectiveness of the additives in the nonpolar lubricant was examined by sliding tribological tests. The friction evolution results on AISI 52100 steel and AISI 316L stainless steel are presented in Figure 1A,B, respectively. During the running-in period, the coefficient of friction (COF) of AISI 52100 steel lubricated by PAO alone starts from 0.13 and decreases to 0.11. Then, the COF begins to increase after 20 m reaching a value of 0.19 at 70 m before gradually declining to 0.14 at the end of the test. Similar trends are observed for PAO-PP and PAO-BMP, in which PP and BMP delayed the increase of the COF to 30 and 110 m, respectively. In the case of PAO-C12, the COF increases during the running-in from 0.10 to 0.12 for 20 m, followed by a slight decrease until the end of the test. By comparing PAO, PAO-PP, and PAO-BMP lubricants, it is worth noticing that the order of COF from low to high is PAO-BMP, PAO-PP, and PAO during the whole duration of the test. Compared to PAO, PAO-PP, and PAO-BMP, the COF of PAO-C12 is the highest at the beginning of the test and the lowest at the end of the test due to the abrupt COF change of PAO, PAO-PP, and PAO-BMP.

In the case of AISI 316L stainless steel, the friction evolution of PAO alone is characterized by a long running-in period with high friction (0.44) at the start and reaching a steady COF of 0.14 after ca. 50 m until the end of the test. PP in PAO reduces the running-in period drastically, keeping friction slightly lower than PAO alone after running-in. In the case of PAO-BMP, the running-in period decreases, and the friction evolution is steady from the start until the end of the test with a COF similar to PAO-PP. C12 in PAO increases friction to 0.15 and fluctuates during the whole test, indicating unstable friction.

Figure 1C,D shows the influence of the additives on the specific wear rate (SWR) of AISI 52100 steel and AISI 316L stainless steel, respectively. In the case of AISI 52100 steel, the SWR value is 5.70 × 10^–6^ mm^3^/Nm for the PAO base lubricant alone. PP and BMP in the base lubricant reduce the SWR by 7 and 36%, respectively. Meanwhile, C12 shows the lowest SWR with a 49% reduction.

In the case of AISI 316L stainless steel, the SWR value for PAO alone is 1.68 × 10^–5^ mm^3^/Nm (ca. 3 times higher than AISI 52100 steel). Both PP and BMP reduce the SWR of AISI 316L stainless steel, in which BMP gives the lowest reduction of 82% and PP gives a 30% reduction. C12 results in the highest SWR with a value of ca. 3.5 times higher than the PAO base lubricant alone.

**Figure 2:**
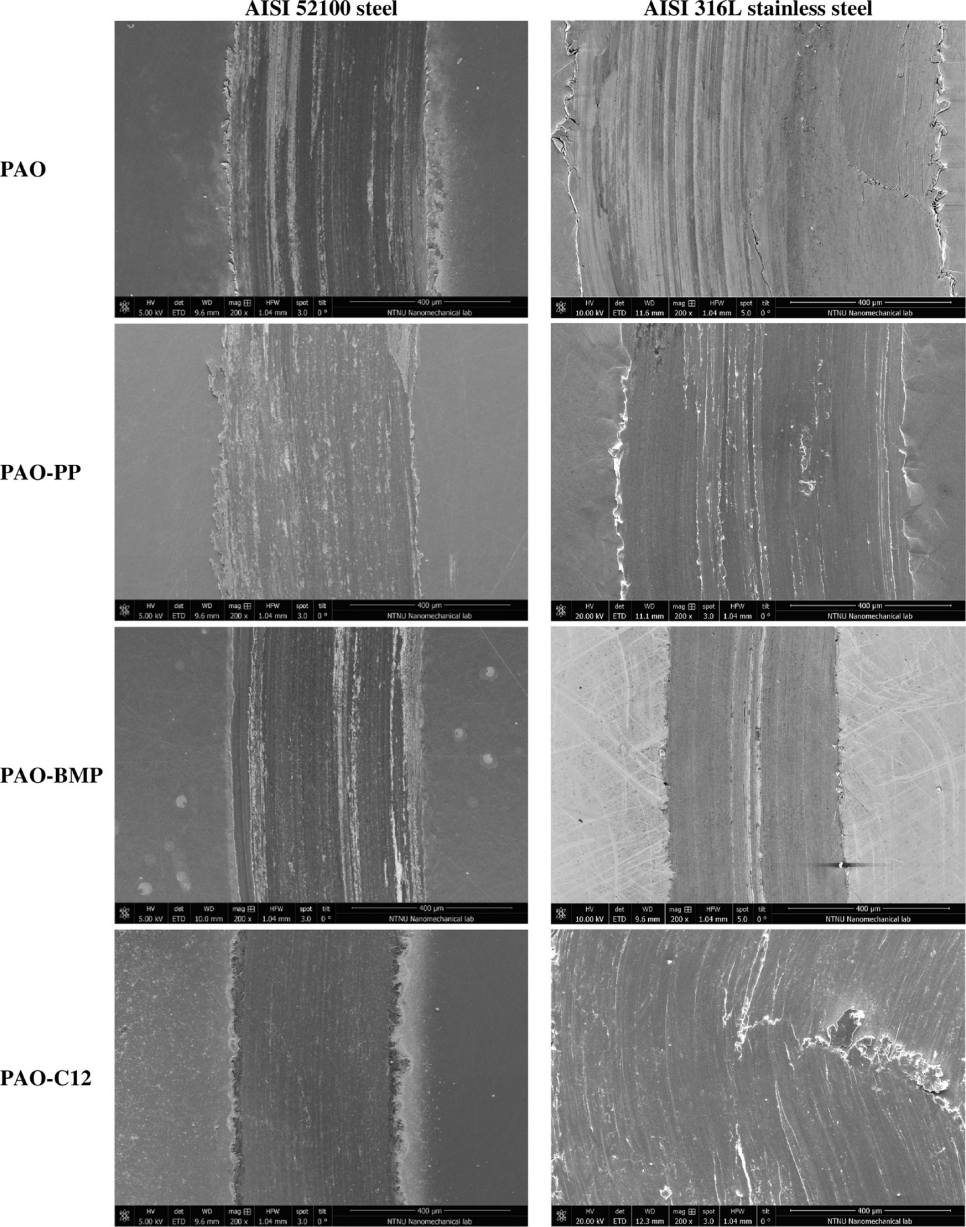
SEM images of the wear tracks of AISI 52100 steel and AISI 316L stainless steel disk tested in different lubricants at room temperature.

Figure 2 shows the SEM images of the wear tracks after testing. The wear track morphology of AISI 52100 steel lubricated by PAO alone shows abrasive wear marks with minor plastic deformation. In the case of PAO-PP and PAO-BMP, the wear tracks of AISI 52100 steel have a similar morphology, in which abrasive wear with minor plastic deformation is observed. On the other hand, a smoother wear surface with no plastic deformation is observed on AISI 52100 steel lubricated by PAO-C12, which is in agreement with the friction evolution and wear results. The wear morphology of AISI 316L stainless steel lubricated by PAO shows a smooth wear surface with signs of abrasive wear and plastic deformation.

In the case of AISI 316L stainless steel lubricated by PAO-PP, the wear track shows plowing with signs of plastic deformation and delamination in some areas. On the other hand, PAO-BMP shows a smooth surface and abrasive grooves with no signs of plastic deformation. PAO-C12 shows delamination, wear flakes, and severe plastic deformation inside the wear track.

**Figure 3:**
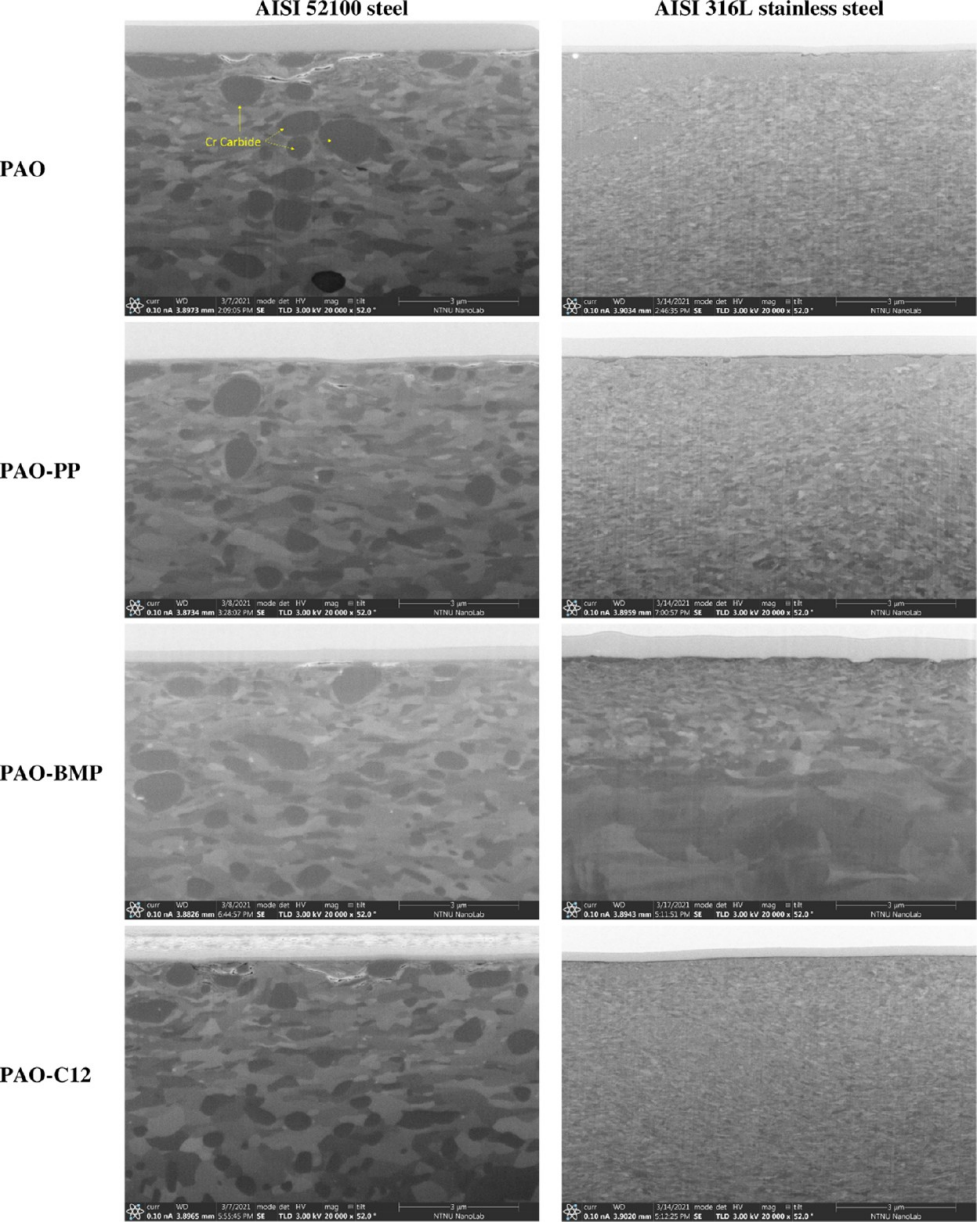
FIB cross-sectional images of the wear tracks of AISI 52100 steel and AISI 316L stainless steel disk tested in different lubricants at room temperature.

The cross-sectional images of the wear tracks prepared by FIB are shown in Figure 3. Double Pt protective layers are visible in the images. The cross-sectional images were recorded at the center of the wear track and perpendicular to the sliding direction. The microstructure of all AISI 52100 steel samples consists of deformed grains with chromium carbide (dark round particles). AISI 52100 steel lubricated by PAO, PAO-PP, and PAO-BMP shows a similar degree of recrystallization and plastic deformation. AISI 52100 steel lubricated by PAO-C12 shows a lower degree of recrystallization and plastic deformation.

AISI 316L stainless steel shows a higher degree of recrystallization and plastic deformation. AISI 316L stainless steel lubricated by PAO, PAO-PP, and PAO-C12 shows a very fine recrystallized area along the cross section. Moreover, the top microstructures underwent severe recrystallization for PAO, resulting in nanometer-size grains. AISI 316L stainless steel lubricated by PAO-BMP shows a lower degree of recrystallization with a thinner deformed region, indicating lower shear strain at the subsurface region.

### Tribofilm Characterization

**Figure 4:**
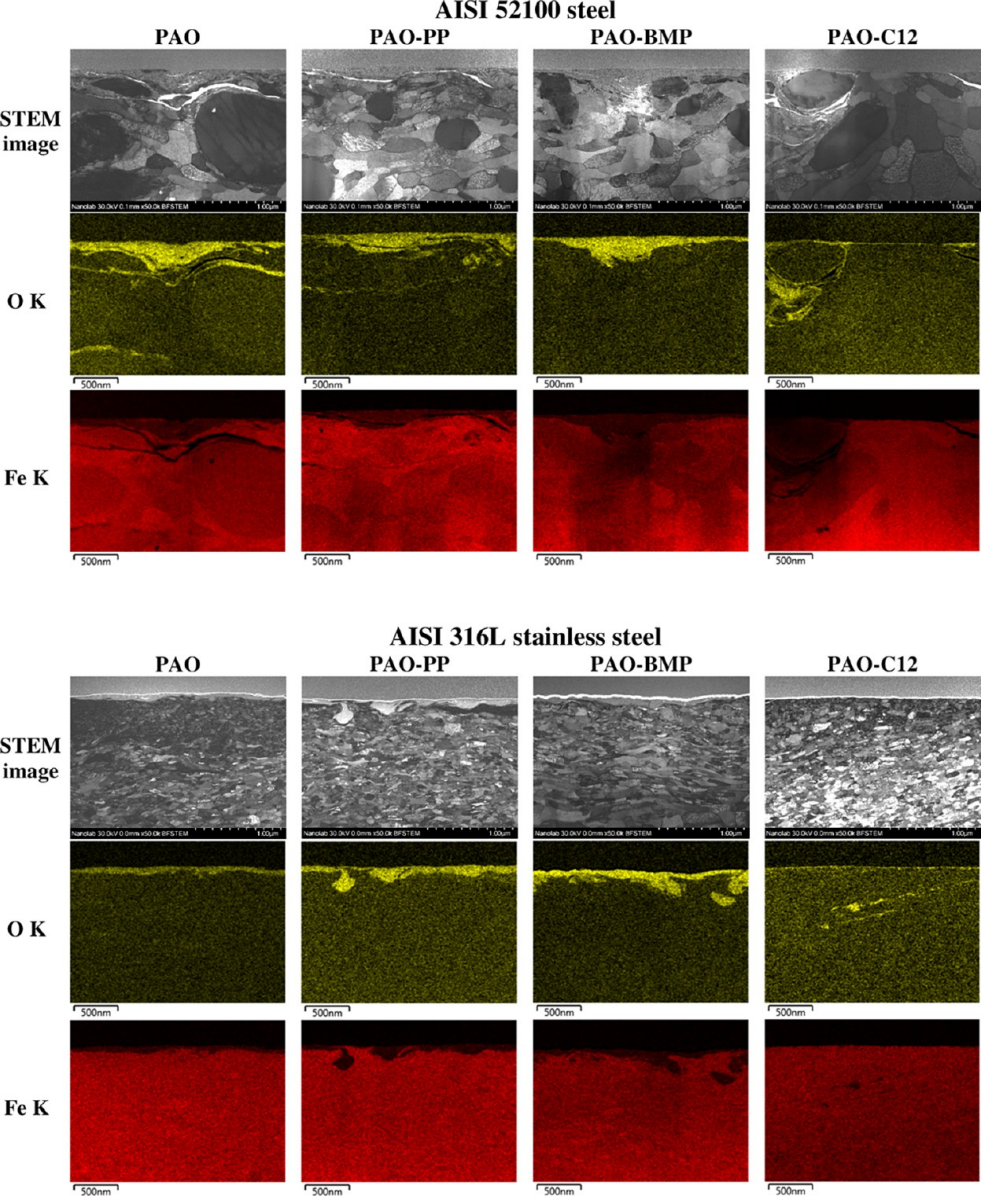
STEM cross-sectional images and EDS elemental mapping of the wear tracks on AISI 52100 steel and AISI 316L stainless steel tested in different lubricants at room temperature.

STEM was used to investigate the tribofilms formed on all samples. The STEM images and EDS elemental mapping are shown in Figure 4 for AISI 52100 steel and AISI 316L stainless steel samples. The chosen elements for mapping were oxygen and iron. The oxygen elemental mapping reveals the presence of oxides in the tribofilm. Phosphorous (P) and fluorine (F) are not shown in the elemental mapping because (1) the Pt Mα and P Kα peaks’ energies are too close, resulting in poor contrast between the platinum protective layer and the phosphorous inside the tribofilm and (2) the overlapping of the F Kα and Fe Lα peaks’ energies make it difficult to distinguish these peaks.

The AISI 52100 steel samples lubricated by PAO, PAO-PP, and PAO-BMP show a thick tribofilm on the surface with a thickness of ca. 50–250 nm (Figure 4). In addition, subsurface cracks are observed for PAO and PAO-PP samples; however, no visible subsurface cracks are observed for PAO-BMP. PAO-C12 shows a thin tribofilm (ca. 15 nm), and cracks are observed in the vicinity of the carbides and on the surface. In the case of AISI 316L stainless steel lubricated by PAO alone, a tribofilm is built on the surface (50–100 nm thickness, Figure 4). Thicker tribofilms are observed for PAO-PP (100–200 nm) and PAO-BMP (100–300 nm). No visible subsurface cracks are observed for AISI 316L stainless steel samples lubricated by PAO, PAO-PP, and PAO-BMP. PAO-C12 shows a thin tribofilm (ca. 15 nm) with signs of oxides trapped deeper into the subsurface region.

EDS point analysis was performed in each sample to obtain a detailed chemical tribofilm composition (Table 3). In the case of all AISI 52100 steel samples, the tribofilm consists of iron and oxygen. Chromium is not detected in the tribofilm because it forms stable chromium carbide. No phosphorous is detected for the PAO-PP sample, indicating that the ILs did not react with the worn surface. In the case of AISI 316L stainless steel samples, the tribofilm consists of iron, chromium, nickel, and oxygen for PAO-C12, and the same elements together with phosphorous for PAO-PP and PAO-BMP samples. Iron, chromium, and nickel originate from the stainless steel material, whereas phosphorous comes from the IL structure, indicating a reaction between the IL and the worn surface.

**Table 3:** EDS Chemical Composition Analysis of the Tribofilms (atom %)

			elemental concentration in the tribofilm (atom %)				
sample	lubricant	tribofilm thickness (nm)	Fe	Cr	Ni	O	P
AISI 52100 steel	PAO	50–250	46.49	0		53.51	
	PAO-PP	50–250	48.87	0		51.13	0
	PAO-BMP	50–250	44.60	0		55.40	0
	PAO-C12	15	40.31	0		59.69	
AISI 316L stainless steel	PAO	50–100	31.06	8.23	3.46	57.25	
	PAO-PP	100–200	25.59	5.79	3.02	60.36	5.24
	PAO-BMP	100–300	21.54	5.18	2.74	69.91	0.63
	PAO-C12	15	37.92	10.42	5.28	46.38	

### Adsorption Study of Additives on Iron- and Stainless Steel-Coated Sensors

**Figure 5:**
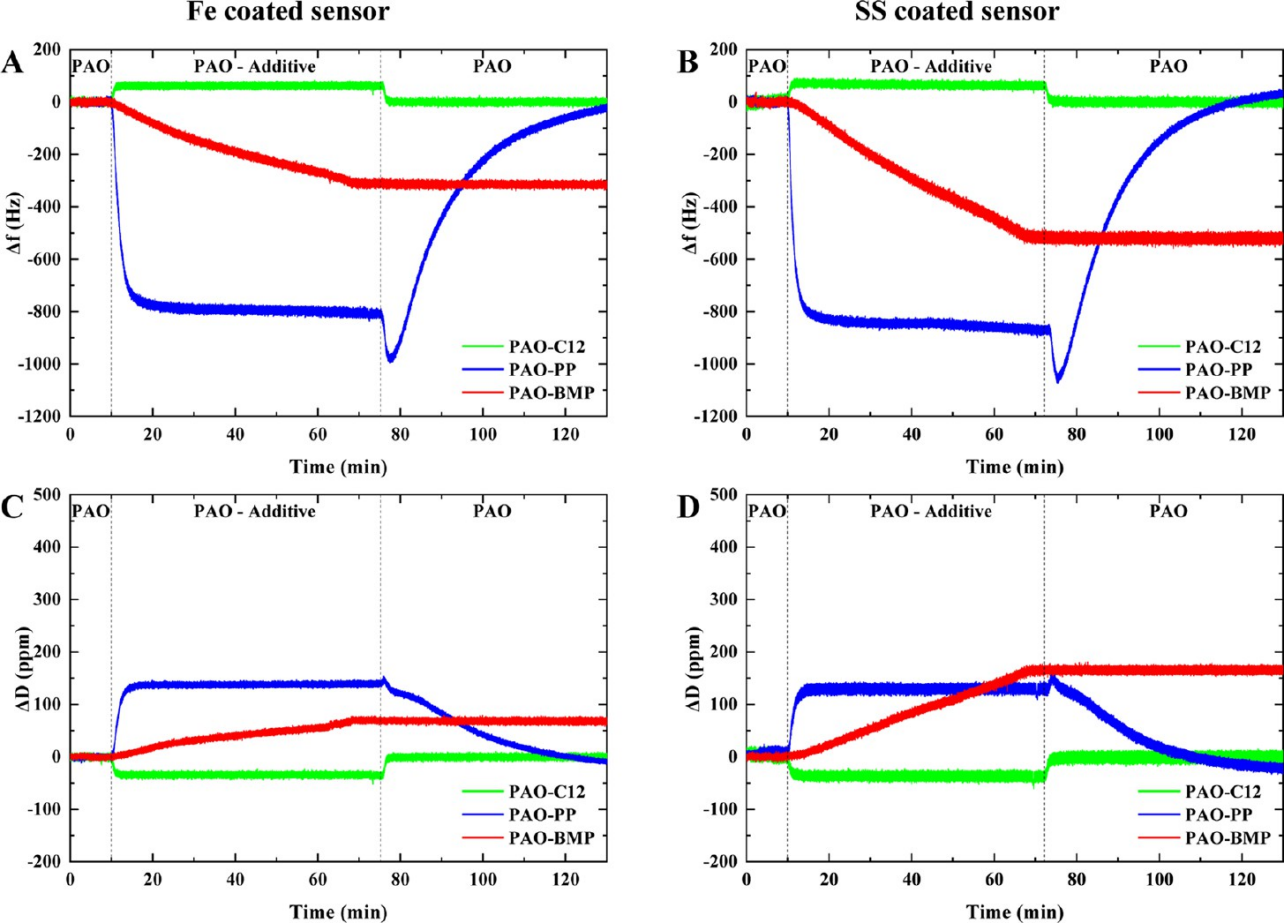
Frequency and dissipation evolution during QCM testing using (A, C) Fe- and (B, D) SS-coated sensors at room temperature.

QCM analysis was performed to study the adsorption of the lubricant additives on iron (Fe)- and stainless steel (SS)-coated sensors. Figure 5 shows the evolution of frequency and dissipation during 10 min of introduction of the base lubricant, followed by 1 h of injection of the formulated lubricant and 1 h of rinsing with the same base lubricant. Note that only the fundamental frequency could be obtained (see the Testing and Characterization Methodssection). By comparing the frequency and dissipation evolution, three phenomena are found: (1) the adsorption kinetics, (2) the initially adsorbed layer, and (3) the strongly adsorbed layer after rinsing. The first 10 min act as a reference for further changes in frequency and dissipation. During the introduction of the formulated lubricant, the frequency slope shift represents the adsorption kinetics, and the frequency shift represents the initially adsorbed layer. The additive can be physically and chemically adsorbed to the QCM sensor’s surface during this process. During rinsing the base lubricant, the adsorbed additive can still stay on the surface or can be partially or completely removed from the surface depending on the bonding strength. The frequency shift during the rinsing defines the bonding strength of the adsorbed layer on the surface.

The frequency and dissipation shifts, shown in Figure 5, indicate utterly different adsorption behaviors for all additives. PP shows higher slope and frequency shift than BMP, indicating faster adsorption kinetics and a larger amount of the initially adsorbed layer on the surface; however, no strongly adsorbed layer is observed after rinsing. By comparing the adsorption on Fe- and SS-coated sensors, PP has slightly faster adsorption kinetics and a slightly higher initially adsorbed layer on the SS-coated sensor. BMP shows strong adsorption and remains on the surface after rinsing despite having slow adsorption kinetics and a low initially adsorbed layer. By comparing the adsorption on Fe- and SS-coated sensors, BMP has faster adsorption kinetics, a higher initially adsorbed layer, and a strongly adsorbed layer after rinsing on the SS-coated sensor. On the other hand, the frequency and dissipation shifts of C12 show opposite behavior to PP or BMP, with a frequency shift toward positive values and a dissipation shift toward negative values. After rinsing, the frequency and dissipation return back to the reference values, indicating complete removal of the adsorbed layer. Similar adsorption behavior is observed for C12 on both Fe- and SS-coated sensors.

## Discussion

### Additives’ Adsorption on the Tribosurface

**Figure 6:**
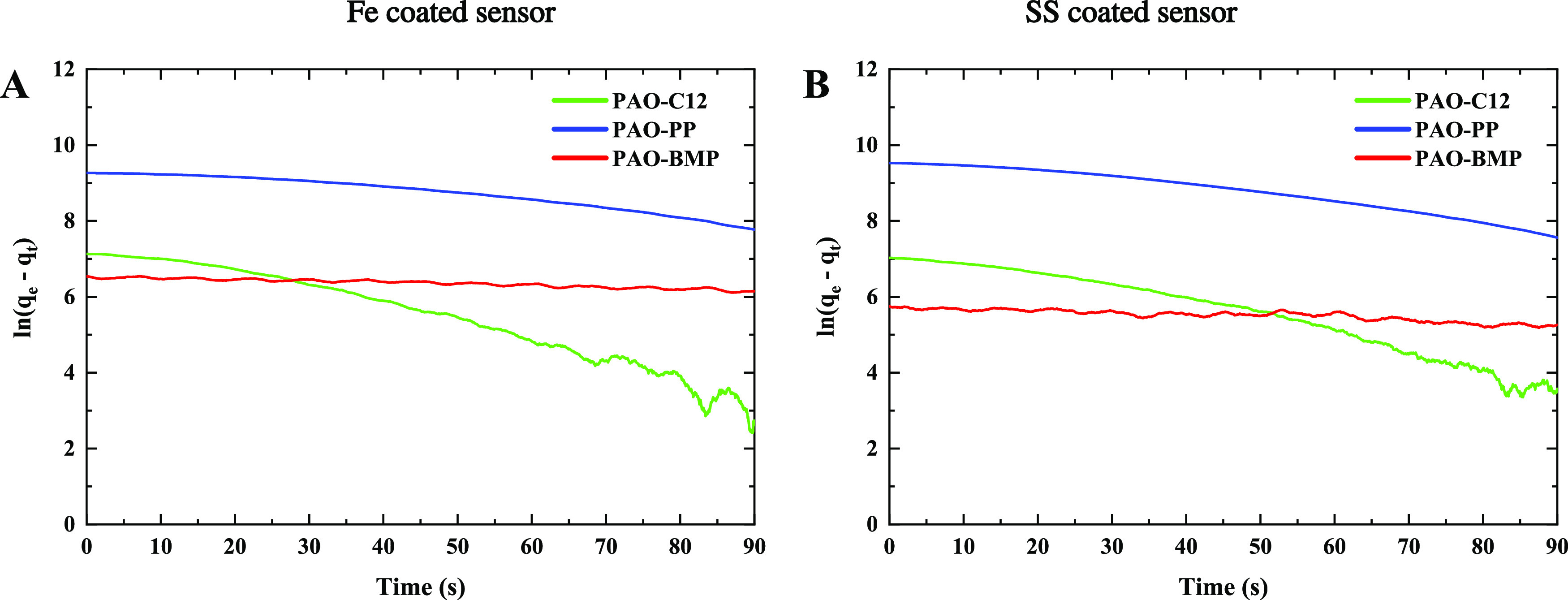
Adsorption kinetics of the additives on the surface of (A) Fe- and (B) SS-coated sensors.

The adsorption study performed with QCM showed different responses in the adsorption behavior of the additives. While the QCM measurements are conducted in static conditions, the tribological test conditions are dynamic where the adsorbed species are continuously removed from the contact surface due to the rubbing action. For the additives to have an effect on the tribological performance, the additive species need to replenish the tribosurface, which is controlled by the adsorption behavior of the additives. During the rubbing action, the adsorbed species are continuously removed from the contact area of the tribosurface. At the same time, electrons are emitted from the worn area, leaving a positively charged surface that attracts the additives to replenish the contact surface again.^23^ Thus, the readsorption of the additive species plays an important role in the tribological behavior. A first-order Langmuir adsorption rate equation can be used to model the adsorption kinetics of the additives as follows^48,49^
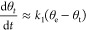
2where *k*_1_ is the first order of the adsorption kinetic constant, and *θ*_e_ and *θ*_*t*_ are the occupied adsorption sites at equilibrium and time *t*. By integrating eq 2, the adsorption kinetic constant is obtained by plotting the dependency of the amount of adsorbed mass versus time as follows
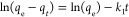
3where *q*_e_ and *q*_*t*_ are the masses adsorbed at equilibrium and time *t*. The logarithmic dependency of the amount of the adsorbed mass versus time is shown in Figure 6 for the first 90 s of adsorption. As shown in eq 3 and Figure 6, the slope of the graph represents the adsorption kinetic constant. By applying linear regression, the adsorption kinetic constants are obtained. For the Fe-coated sensor, the adsorption kinetic constants are 0.0470, 0.0164, and 0.0041 s^–1^ for C12, PP, and BMP, respectively. In the case of the SS-coated sensor, the adsorption kinetic constants are 0.0411, 0.0218, and 0.0052 s^–1^ for C12, PP, and BMP, respectively. Thus, on both sensors, C12 holds the fastest adsorption kinetics followed by PP and BMP.

The relationship between the frequency shift and the mass change of the QCM sensor was first proposed by Sauerbrey in the following Sauerbrey equation^50^
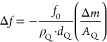
4where Δ*f* and Δ*m*/*A*_Q_ are frequency shift (Hz) and mass change per unit area (kg/m^2^). *f*_0_, ρ_Q_, and *d*_Q_ are the natural frequency in vacuum (Hz), density (kg/m^3^), and thickness (*m*) of the quartz plate, respectively. Note that Sauerbrey performed his experiments under vacuum; therefore, the Sauerbrey equation is the basis for using QCM to measure adsorption in vacuum or gas phase regardless of the viscoelastic properties of the adsorbate.

Due to their ionic nature and their high dipole moment, ILs have a higher tendency to be adsorbed on the metallic surface to form a single- or multilayer structure.^35^ Using the Sauerbrey equation, the adsorbed layer structure can be predicted. In the case of PP, one molecule of PP has a length of 2.03 nm (from the structure analysis using MarvinSketch software), which gives a frequency shift of −9.34 Hz for one adsorbed layer. Using the same procedure for BMP (the length of one molecule is 2.11 nm), the calculated frequency shift is −15.93 Hz for one adsorbed layer. As shown in Figure 5, the frequency shift of PP and BMP on the Fe-coated sensor are −820 and −320 Hz, and on the SS-coated sensor, they are −877 and −525 Hz, respectively. Thus, it is expected that PP and BMP will form multilayer structures on both Fe- and SS-coated sensors. PP will have a thicker adsorbed layer compared to BMP. Generally, ILs in nonpolar media, such as PAO, exhibit no ionic dissociation, maintaining the dipole moment of the molecule. As shown in Figure 5, BMP stays on the surface of the sensor after rinsing with PAO, indicating strong adsorption of BMP to the sensor surface. The strong adsorption of BMP indicates a chemisorption process, which promotes the formation of a tribofilm. The presence of high electronegative atoms (F) promotes strong adsorption to the metallic surface. Similar behavior was observed for BMP in water–glycol,^39^ confirming that the high electronegative atoms play a role in the adsorption process and the further tribofilm formation. On the other hand, PP weakly adsorbs to the surface, showing that it undergoes a physisorption process. The anion of PP has a smaller density of negative charge than BMP and its smaller size generates a weaker interaction with the metal surface. Figure 5 shows that the stainless steel surface tends to adsorb more ILs, probably due to the different surface chemistry (i.e., stainless steel creates a nanometric passive film of chromium oxide on the surface). In the case of C12, a positive but low frequency shift was observed, indicating a thinner adsorbed layer than the ILs. In addition, complete removal of the adsorbed layer after rinsing with PAO indicates a physisorption process (Figure 5).

A simulation approach has been used to understand the adsorption of C12 on Fe- and SS-coated sensors. In 1999, Voinova et al. proposed a continuum mechanics approach to describe the adsorption of a layered polymer film in a liquid environment.^51^ There are several theoretical methods for quantitative interpretation of the viscoelastic response of QCM data, such as continuum mechanics, electrical circuit, and transmission line analysis methods.^52,53^ However, only the continuum mechanics approach directly links the QCM data to the adsorbed layer’s physical description, which correlates the measured frequency and dissipation with the adsorbed layer properties. Using Voinova’s approach, the viscoelastic properties of an adsorbed layer with an arbitrary thickness covering the surface of a quartz sensor immersed in liquid can be analyzed. The Voigt element was used to model the viscoelastic material in this approach. Spring and dashpot in a parallel arrangement represent the shear elasticity modulus (μ) and the shear viscosity coefficient (η) of the layered film, respectively. Assumptions for the adsorbed layer are rigidly attached (no slip), evenly distributed, and homogeneous thickness, density, viscosity, and elasticity properties.

**Figure 7:**
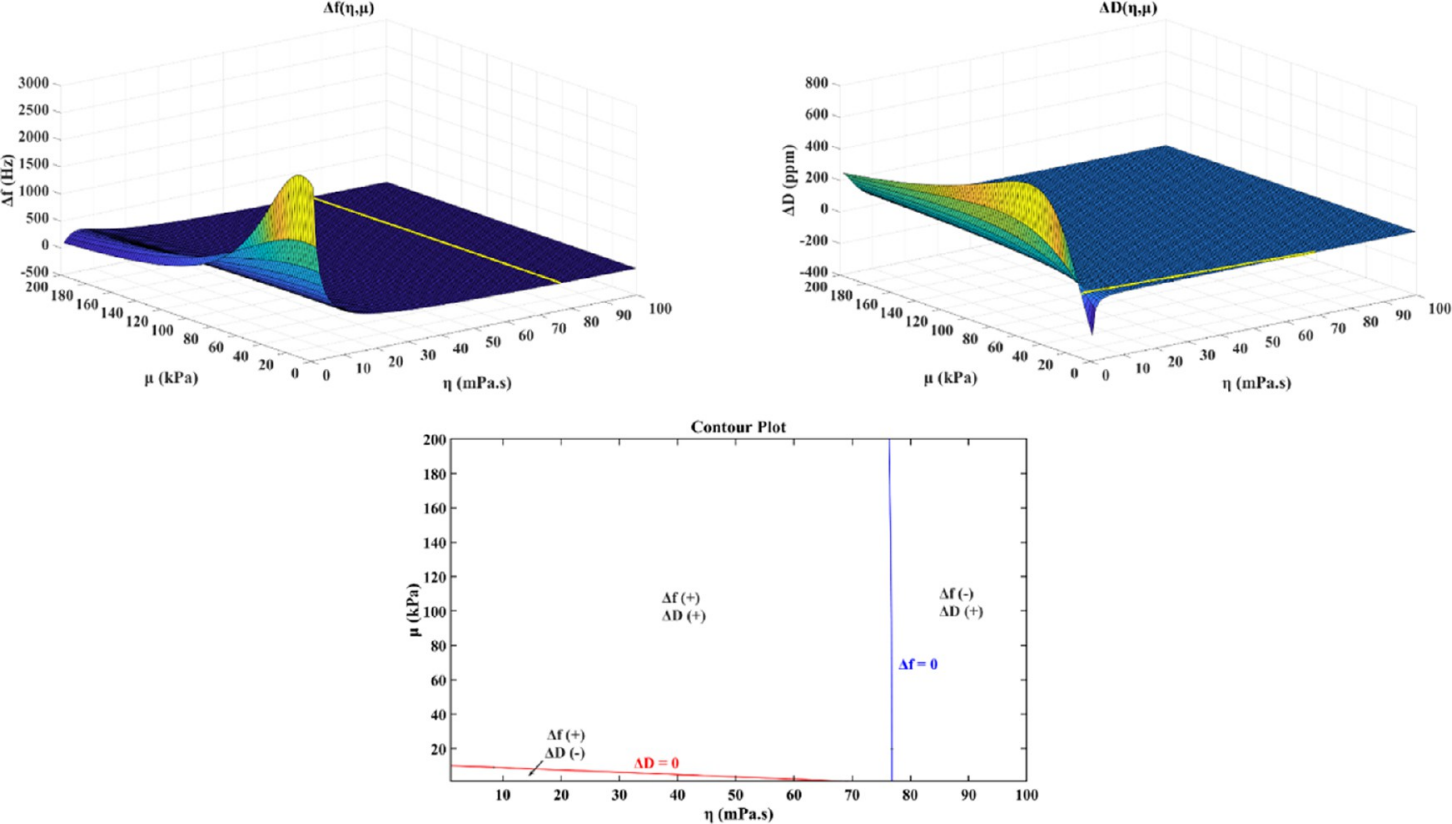
Numerical simulation of the frequency shift (A) and the dissipation shift responses (B) as a function of the shear elasticity modulus and the shear viscosity coefficient of the thin viscoelastic C12 layer. A contour plot is taken from (A) and (B), showing the different regions of Δ*f* and Δ*D* values (C).

The acoustic response of the QCM was modeled with MATLAB varying the viscoelastic layer properties (η and μ) using the general solution of the wave equation for the thin viscoelastic layer immersed in a bulk liquid. Figure 7 shows the result of the numerical simulation of the model for the thin viscoelastic C12 layer of a thickness of 10 nm and a density of 880 kg/m^3^ when the sensor oscillates with a frequency of 5 MHz in PAO (ρ = 840.8 kg/m^3^, η = 83.13 mPa·s). A C12 thickness of 10 nm for the analysis was selected based on the thickness measured using atomic force microscopy (AFM).^40^ In Figure 7A,B, the zero value line (yellow line) is presented, forming the basis for constructing Figure 7C.

To explain the acoustic response in Figure 7, the following simplified equations are provided by considering one thin viscoelastic layer under bulk liquid and keeping only the first-order approximation^51^
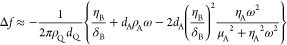
5
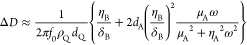
6
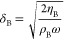
7
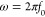
8where ρ_B_ and η_B_ are the density (kg/m^3^) and viscosity (Pa·s) of bulk liquid, whereas *d*_A_, ρ_A_, μ_A_, and η_A_ are the thickness (m), density (kg/m^3^), shear elasticity modulus (Pa), and shear viscosity coefficient (Pa·s) of the adsorbed layer, respectively. In eq 5, the resonance frequency shift depends on the bulk liquid term (η_B_ and δ_B_), and the difference between the layer mass contribution (*d*_A_, ρ_A_, and ω) and layer viscoelastic contribution (μ_A_ and η_A_), whereas in eq 6, the resonance dissipation shift depends on the bulk liquid term *η*_B_/δ_B_ and layer viscoelastic contribution (μ_A_ and η_A_). Note that eqs 5 and 6 take the reference from vacuum, meaning the bulk liquid contribution is relative to vacuum. In a liquid medium, the contribution of the reference liquid should be included in the equations as follows
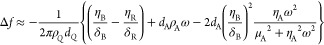
9
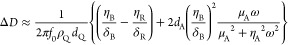
10
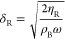
11where ρ_R_ and η_R_ are the reference liquid’s density (kg/m^3^) and viscosity (Pa·s), respectively. In the case of similar density and viscosity between reference and bulk liquids, the influence between these two can be canceled
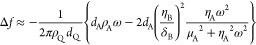
12
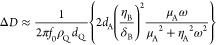
13It is worth noticing that due to the approximation, the layer mass contribution is neglected in eq 13. However, from the simulation in Figure 7B (without approximation), there is a region with a negative dissipation value when the layer has a low shear elasticity modulus with the shear viscosity lower than the shear viscosity of the bulk.

**Figure 8:**
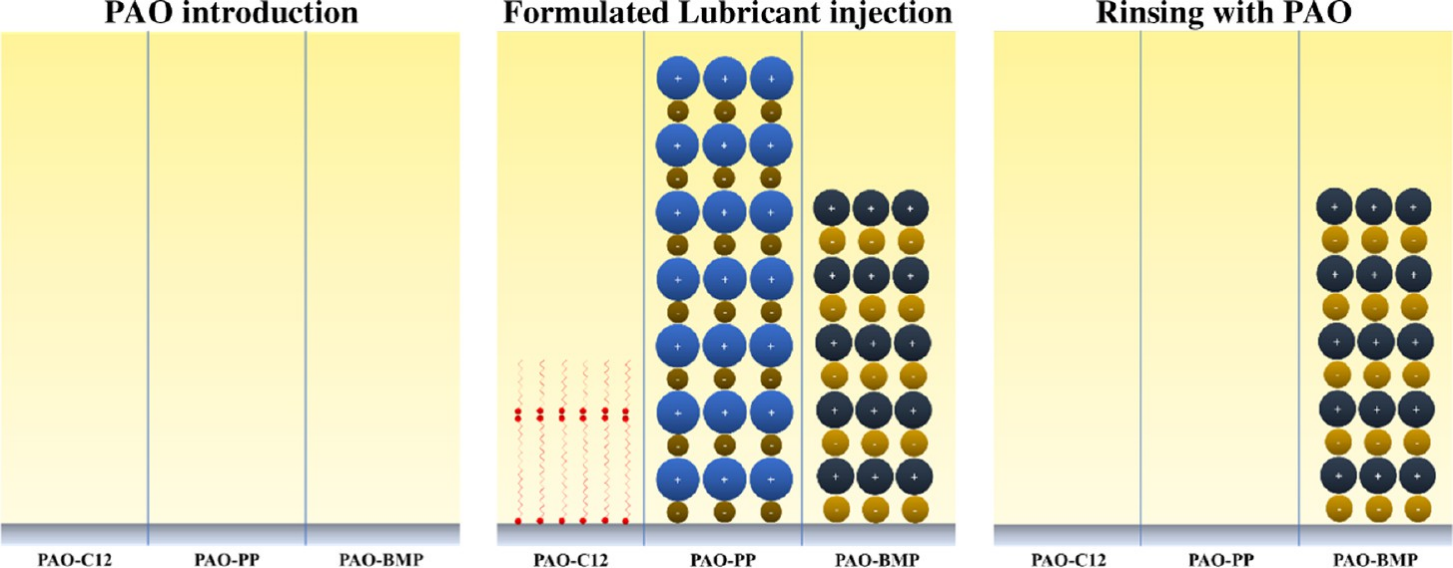
Molecular assemblies’ illustration of the additives on both the Fe and SS surfaces during the QCM tests.

From the simulation results and according to eq 12, the unexpected positive frequency shift (and negative dissipation shift) found experimentally for C12 in Fe- and SS-coated sensors (Figure 5) is a result of the layer viscoelastic contribution rather than the adsorption mass contribution. The C12 adsorbed layer has both low shear viscosity and a low shear elasticity modulus compared to the bulk liquid (PAO), thus resulting in a positive shift of the frequency and a negative shift of the dissipation (Figure 7C). Therefore, the negative frequency shift of PP and BMP additives is mostly influenced by the adsorption mass contribution. Thus, the adsorbed layer of PP and BMP must have high shear viscosity and a high shear elasticity modulus compared to the bulk liquid (PAO). All of the adsorption responses are summarized in Table 4, and the molecular assemblies expected on the surface of the sensors after QCM testing and simulation results are illustrated in Figure 8.

**Table 4:** Summary of the Adsorbed Layer Properties on Both Fe- and SS-Coated Sensors

additive	adsorption kinetics	initially adsorbed layer	strongly adsorbed layer	mass layer contribution	shear viscosity (compared to bulk liquid)	shear elasticity modulus
SS-PP	medium-high	very high	no	very high	high	high
Fe-PP	medium	high	no	high	high	high
SS-BMP	slow	medium-high	yes	medium-high	high	high
Fe-BMP	very slow	medium	yes	medium	high	high
SS-C12	very fast	very low	no	very low	low	low
Fe-C12	very fast	very low	no	very low	low	low

### Effect of Lubricant Formulation on the Tribofilm Chemical Composition

PAO-PP and PAO-BMP promote noticeable tribofilm formation on both surfaces, as shown in Figure 4. Interestingly, PAO alone creates a thick tribofilm, indicating that only the presence of oxygen in the lubricant is enough to contribute to a tribochemical reaction leading to tribofilm growth. In the case of PAO-C12, a thin tribofilm is formed, indicating that C12 hinders tribofilm growth by occupying the active sites for the tribochemical reaction. Tribofilm formation consists of three stages, i.e., tribofilm initiation, wear debris generation and breakdown, and tribofilm growth.^54^ For tribofilm initiation, the reactive elements inside the lubricant, such as oxygen, ILs, and ILs’ decomposition products, adsorb to and react with the nascent surface to form a thin interlayer film. This interlayer can act as a good bonding layer between the metallic substrate and the tribofilm or, on the other hand, it can act as a barrier layer hindering tribofilm growth. The mechanical action will generate wear debris at the contact zone. Some wear debris may get trapped and broke down to nanosize in the contact zone, and others may be wiped away. The nanosized wear debris will consequently react with reactive elements in the lubricant due to the thermomechanical process in the contact area. Both unreacted and reacted wear debris are deposited and smeared on the tribosurface (which is occupied by an interlayer film from tribofilm initiation), thus leading to further film growth.

**Figure 9:**
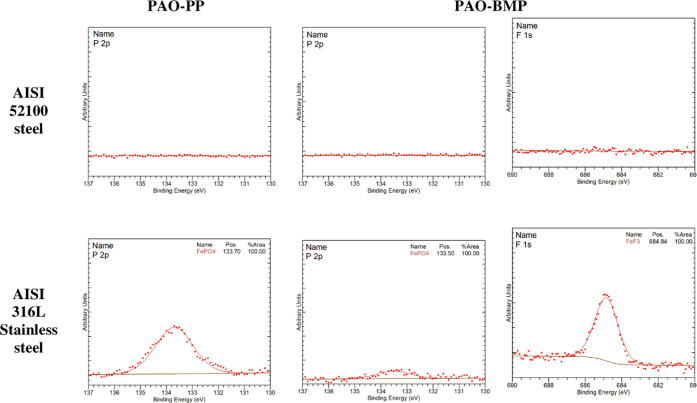
P and F XPS spectra inside the tribofilm of AISI 52100 steel and AISI 316L stainless steel lubricated by PAO-PP and PAO-BMP (taken after 85 s sputtering time).

XPS was used to study the tribofilm chemical composition of the tribofilms on both the surface and the subsurface. For surface analysis, the wear track was sputtered with argon for 5 s to remove the contamination before the XPS data acquisition. For subsurface analysis, the wear track was sputtered for 85 s (going deeper in the tribofilm thickness). Surface and subsurface analysis revealed the same chemical composition. The detailed XPS spectra for subsurface analysis are shown in Figure 9. No P or F was detected in the tribofilm of AISI 52100 in any of the lubricants formulated with ILs. In the case of AISI 316L stainless steel, P is detected in the tribofilms of both PP and BMP. XPS analysis further found that P created an iron phosphate phase. In addition, F in the form of iron fluoride was found for BMP. The XPS results have confirmed that the adsorption of the ILs did not result in any chemical reaction with AISI 52100 steel, but they did react with AISI 316L stainless steel. This confirms that the surface chemistry of the moving parts influences the tribofilm formation given the same lubricant composition. Interestingly, it was found by QCM that the adsorption kinetics and the initially adsorbed layer of the ILs were larger on SS than those on Fe-coated sensors (Table 4 and Figure 6).

### Tribofilm and Adsorbed Layer Effects on Friction

As shown in Figure 1A, there is a transition in the friction evolution of AISI 52100 steel lubricated by PAO, PAO-PP, and PAO-BMP, which results in higher friction than PAO-C12. High friction, together with the high initial Hertzian contact pressure (1.96 GPa), results in a higher degree of recrystallization and plastic deformation (Figure 3). As shown in Table 3, thick tribofilms are formed for PAO, PAO-PP, and PAO-BMP, whereas a thin tribofilm is formed for PAO-C12. Therefore, the formation of thick tribofilms can increase friction. This phenomenon has also been observed when using ZDDP, a well-known antiwear additive. Dawcyzk et al. showed that an increase in friction is a direct result of the increase in the effective roughness of the worn surfaces due to tribofilm formation.^55^ As shown in Figure 2, PAO, PAO-PP, and PAO-BMP create rougher worn surfaces than PAO-C12 due to thicker tribofilm formation.

**Figure 10:**
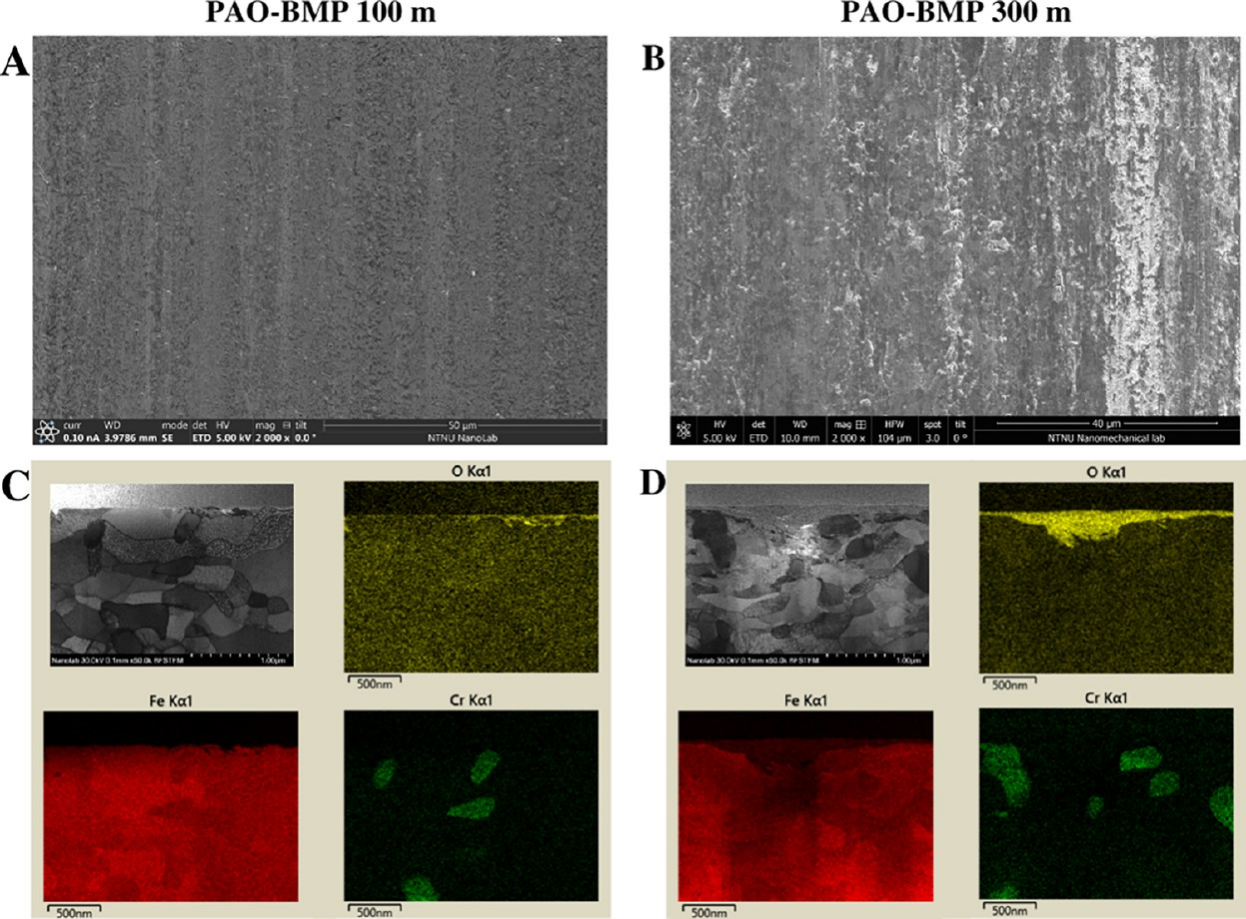
Top view and STEM image with elemental mapping of the AISI 52100 steel worn surface lubricated by PAO-BMP tested for 100 m (A, C) and 300 m (B, D).

To further understand the influence of tribofilm and surface roughness on friction, a shorter test was performed for PAO-BMP on AISI 52100 (terminated after 100 m sliding, right before the friction transition seen in Figure 1A). The top view of the worn surface and the STEM image with elemental mapping of PAO-BMP 100 m are shown in Figure 10 along with PAO-BMP 300 m sample for comparison. The PAO-BMP 300 m wear track shows a rougher surface, whereas a smoother surface is observed for PAO-BMP 100 m due to thin tribofilm formation. These wear surface morphologies are in agreement with the friction evolution of PAO-BMP on AISI 52100 steel in Figure 1A, in which a smoother surface results in lower friction and vice versa. Therefore, in the case of AISI 52100 steel, a thick oxide tribofilm is responsible for high friction.

However, a friction increase due to the thick tribofilm is not observed for AISI 316L stainless steel. As shown in Figure 1B, the friction evolution of AISI 316L stainless steel lubricated by PAO, PAO-PP, and PAO-BMP shows low values even though a thick tribofilm was formed, implying that a thick tribofilm might not be the factor playing the most important role in friction for stainless steel. As shown in Table 3 and Figure 9, the formed tribofilm on stainless steel contains oxides and hydroxides from different metals, P and F, whereas the tribofilm on AISI 52100 steel only contains Fe oxides and hydroxides. Therefore, the chemical composition of the tribofilm resulted in a different frictional response.

In addition, the friction behavior is influenced not only by the presence of a tribofilm but also by the adsorbed additives on the tribosurface.^35^ The adsorbed layer properties, such as thickness or mass, viscoelastic properties, and bonding strength, define its friction-reducing ability. Comparing PAO, PAO-PP, and PAO-BMP friction in Figure 1A, the effect of the type of additive on friction is clear, where PAO-BMP gives the lowest friction evolution both before and after tribofilm formation. As shown in Table 4, PP has faster adsorption kinetics and adsorption mass and weak adsorption. BMP shows slower adsorption kinetics and lower adsorption mass but adsorbs strongly to the sensor surface, indicating that the bonding strength plays the most important role in the friction-reducing ability. A strongly adsorbed layer (BMP) maintains the layer’s integrity during the sliding action. In addition, a stronger adsorbed layer produces a more durable layer; thus, BMP maintains lower friction on longer distances than PP. In the case of AISI 316L stainless steel, the adsorbed layer influences the running-in period, in which a durable adsorbed layer (BMP) reduces the running-in period significantly (Figure 1B). This leads to the thinnest recrystallization region, i.e., less severe shear forces (Figure 3).

In the case of PAO-C12, a thin tribofilm and a less durable adsorbed layer were formed both on AISI 52100 steel and AISI 316L stainless steel, but different friction behaviors were still observed. PAO-C12 shows steady and low friction evolution on AISI 52100 steel (Figure 1A); in contrast, higher friction evolution with high fluctuation is observed for AISI 316L stainless steel (Figure 1B). Both tribofilms on AISI 52100 steel and AISI 316L stainless steel are thin oxide layers (Table 3), and the QCM study indicates that C12 formed a less durable adsorbed layer both on Fe- and SS-coated sensors (Table 4). The main difference in this case is the mechanical properties of the substrates, in which AISI 52100 steel has a higher hardness than AISI 316L stainless steel. For the lower hardness material, a less durable adsorbed layer and thin tribofilm cannot withstand the boundary lubricating conditions, resulting in plastic deformation, as shown in Figure 2. On the other hand, the harder material, with a less durable adsorbed layer and a thin tribofilm, can withstand the applied boundary lubricating condition, thus maintaining low friction.

### Tribofilm and Adsorbed Layer Effects on Wear

**Figure 11:**
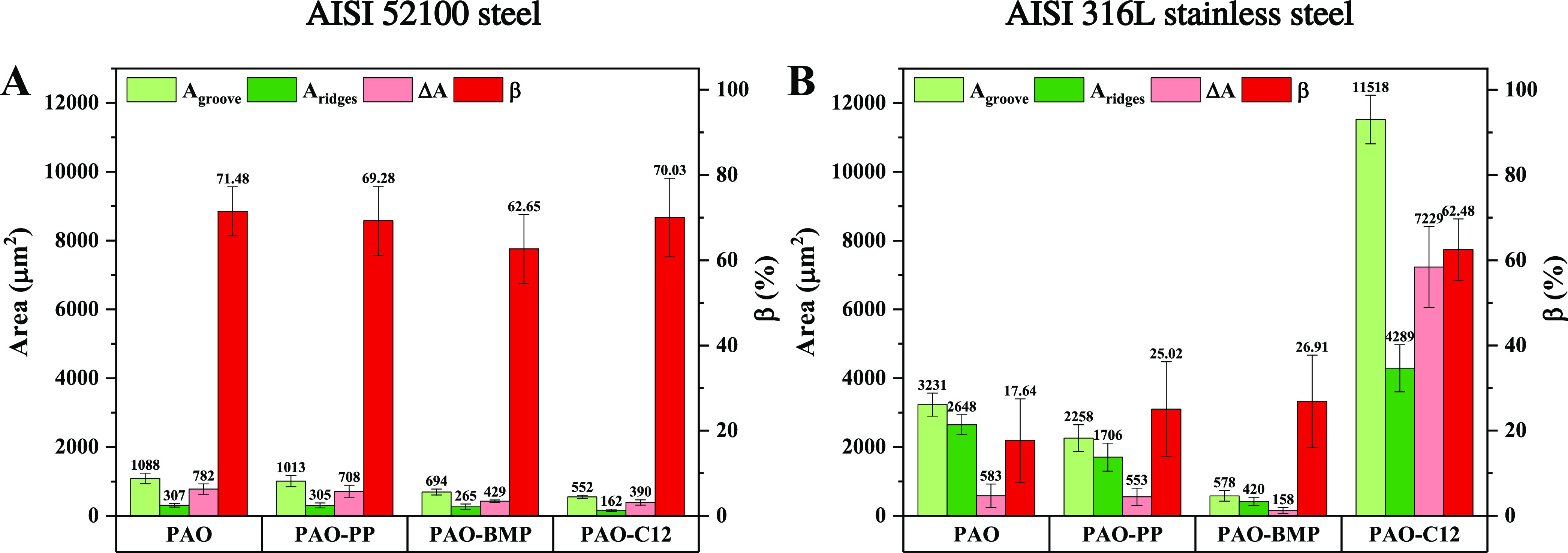
Calculated wear area of AISI 52100 steel (A) and AISI 316L stainless steel (B) lubricated by PAO with and without additives.

A detailed wear analysis was performed by investigating the wear profiles taken by IFM for each tested condition. From the wear profile analysis (not shown in this paper), ridges were formed along the wear track due to material displacement to the sides of the wear track during testing, indicating plastic deformation. From the wear profile, the difference between the area of the ridges (*A*_ridges_) and the area of the wear track groove (*A*_groove_) gives the actual area loss (Δ*A*). The ratio between the area loss and wear track groove is the material loss degree (β) and it is calculated by the following equation^56^
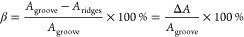
14The β value indicates the wear mechanism, where lower β means more plastic deformation and higher β more abrasive wear. The wear profile analysis results are shown in Figure 11.

In the case of AISI 52100 steel, all additives reduce the area loss (Δ*A*). Interestingly, the β value between all lubricants is comparable (60–70%), indicating a similar abrasive wear mechanism. Similar to the friction behavior, wear is also highly influenced by the presence of a tribofilm and adsorbed layer. As the tribofilms have similar thickness and chemical composition for PAO-PP and PAO-BMP, the wear reduction is mainly affected by the adsorbed layer. A durable adsorbed layer (BMP) gives lower wear. The shorter test performed for PAO-C12 and PAO-BMP (terminated after 100 m sliding, right before the thick tribofilm formation) showed that the area loss Δ*A* for PAO-BMP 100 m (133 μm^2^) was 50% lower than PAO-C12 100 m (56 μm^2^). In addition, the calculated β value for PAO-BMP 100 m (24%) was smaller than for PAO-C12 100 m (39%), indicating more plastic deformation. Therefore, in the absence of a thick tribofilm (100 m tests), a durable adsorbed layer provides better wear resistance.

In the case of AISI 316L stainless steel lubricated by PAO, PAO-PP, and PAO-BMP, a thick tribofilm also forms on the tribosurface, as shown in Table 3. These tribofilms contain metallic oxides and hydroxides of iron, chromium, nickel, and iron phosphates and fluorides, yielding durable tribofilms. Figure 11A shows that PAO, PAO-PP, and PAO-BMP have similar low β values, indicating a similar plastic deformation wear mechanism mostly due to the lower hardness of the substrate. Interestingly the wear of PAO-BMP is the lowest among all tested lubricant–substrate combinations, suggesting that the combination of a durable tribofilm with a durable adsorbed layer significantly reduces wear regardless of the hardness of the substrate. For PAO-C12, abrasive wear was the main mechanism (Figure 11B). Similar to AISI 52100 steel, C12 creates a thin tribofilm and a less durable adsorbed layer. C12 produces higher wear on AISI 316L stainless steel than on AISI 52100 steel, indicating that substrate hardness plays an important role in this case.

### Effect of Steel Hardness and Composition on the Lubricating Mechanisms

**Figure 12:**
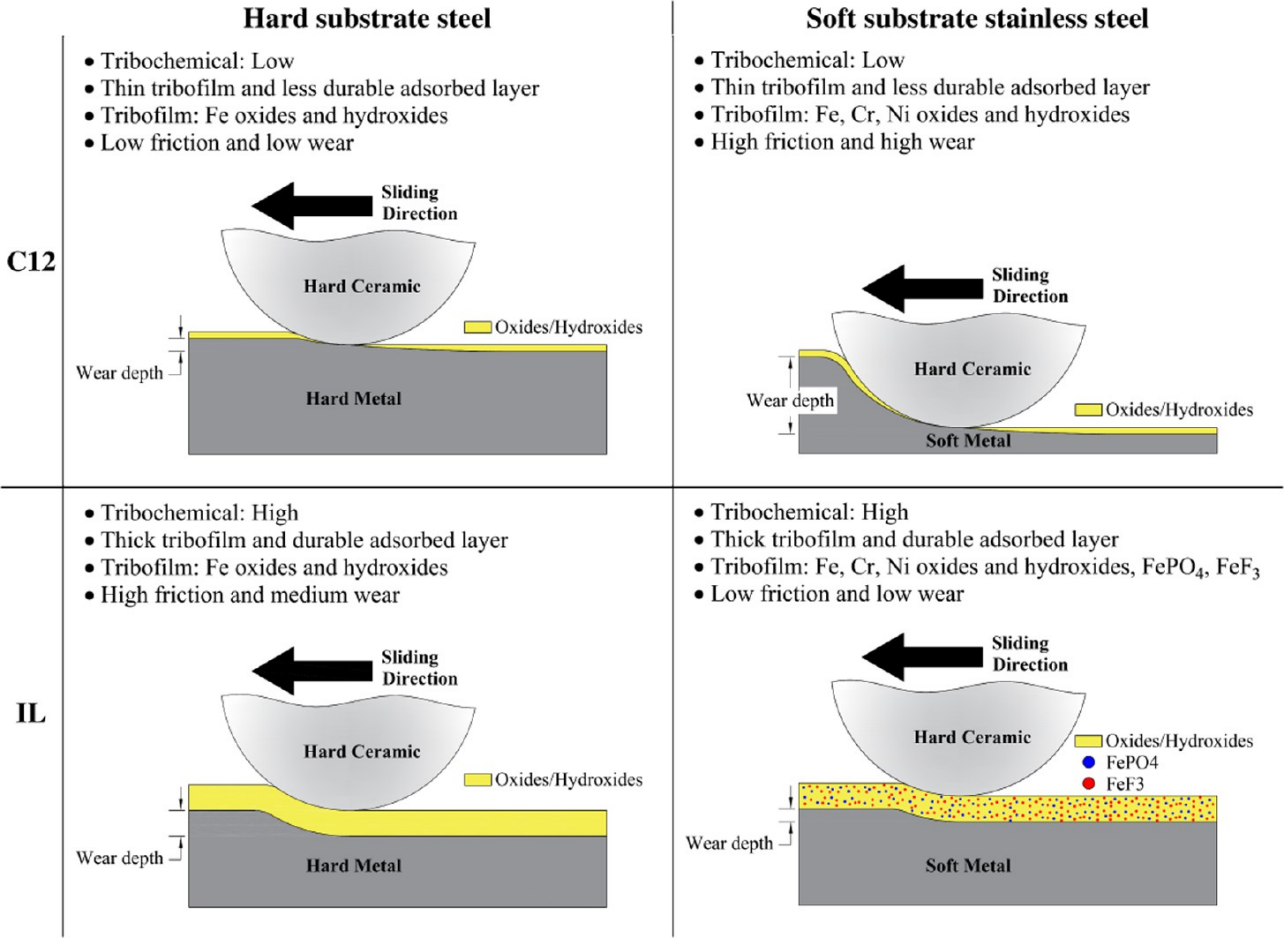
Schematic illustration of the boundary lubricating mechanisms of hard versus soft substrate lubricated by PAO-C12 and PAO-IL.

In this study, it was found that to control friction and wear of the lubricated tribosystems, the mechanical properties and chemical composition of the tribomaterial are as important as the surface adsorption behavior of the additives on the tribosurfaces. Figure 12 illustrates the lubrication mechanisms of C12 and ILs in PAO as a function of tribomaterial hardness and composition. In the case of C12, thin tribofilms and a less durable adsorbed layer were formed on the tribosurfaces; thus, the hardness of the substrate has been the predominant factor affecting the tribological behavior of the system. A thin tribofilm and a less durable adsorbed layer formed on the soft metal substrate (AISI 316L stainless steel) were not strong enough to withstand the boundary lubricating conditions, resulting in severe plastic deformation (Figure 2), which in turn increased the surface roughness leading to high friction and subsequent wear.

In the case of ILs, thick tribofilms and durable adsorbed layers were formed on the tribosurfaces. The tribofilm chemical composition was the predominant factor influencing frictional and wear behavior for the ILs. The tribofilms’ composition was different depending on the steel composition. The tribofilm formed on AISI 316L stainless steel consisted of Fe oxides and hydroxides and the chemical elements associated with the ILs (P and F). The tribofilm formed on AISI 52100 steel consisted of Fe oxides and hydroxides only. These different tribofilms resulted in different friction and wear behaviors. In both cases, the wear rates were at the same order of magnitude; however, the presence of FePO_4_ and FeF_3_ in the tribofilms significantly increased the wear resistance of AISI 316L stainless steel (Figure 11). The main differences between the two steels were found for friction. In the case of AISI 52100 steel, despite forming a durable adsorbed layer, the presence of hard Fe oxides and hydroxides and the lack of F and P in the tribofilm created rougher tribosurfaces leading to high friction. For the AISI 316L stainless steel, the durable adsorbed layer and the presence of FePO_4_ and FeF_3_ in the tribofilm provided low and steady friction.

## Conclusions

The lubricating mechanisms of two ionic liquids as lubricant additives in PAO on AISI 52100 steel and AISI 316L stainless steel have been investigated and compared with dodecanoic acid (C12) as a reference additive. The following conclusions can be drawn from this work

- The adsorption study by QCM-D reveals that C12 formed thin and less durable adsorbed layers on both Fe- and SS-coated sensors. In the case of ILs, two different surface adsorption behaviors were observed: (1) a thick but not strongly adsorbed layer in the case of PP and (2) a thick and strongly adsorbed layer in the case of BMP. The sensor’s chemical composition does not influence the adsorption behavior of C12; however, it influences the adsorption behavior of ILs in which the SS-coated sensor tends to adsorb more ILs than the Fe-coated sensor.
- The tribofilm analysis shows that C12 hinders the tribofilm growth, resulting in thin tribofilm formation; on the other hand, ILs promote noticeable tribofilm formation on both AISI 52100 steel and AISI 316L stainless steel. The chemical composition of the substrate determines the chemical composition of the tribofilm in which the tribofilm formed on AISI 52100 steel consists of Fe oxides and hydroxides. In contrast, the tribofilm formed on AISI 316L consists of Fe, Cr, and Ni oxides and hydroxides and the chemical elements associated with the ILs (P and F). The differences in the chemical composition influence the mechanical properties and durability of the tribofilms.
- C12 showed better tribological performance on AISI 52100 steel than on AISI 316L stainless steel despite it created less durable adsorbed layers and thin tribofilms. The tribomaterial hardness played the most important role in the tribological performance of this additive, where a higher hardness could withstand the boundary lubricating conditions.
- For the ILs, the tribological performance was controlled by tribofilm formation for a high hardness substrate (AISI 52100 steel), where the presence of oxide tribofilms increased friction and wear. In the case of a low hardness substrate (AISI 316L stainless steel), the tribological performance was controlled by both the adsorbed layer properties and the tribofilm durability, where a strongly adsorbed layer led to a durable tribofilm with excellent friction-reducing and antiwear performance.
- BMP on AISI 316L stainless steel showed the best tribological performance of all tests due to the formation of a durable adsorbed layer and a strong tribofilm. The durability of the BMP adsorbed layer was a result of the high electronegativity of fluorine atoms, whereas the durability of the tribofilm was a result of the tribochemical reaction of BMP with the tribosurface, resulting in the formation of FePO_4_ and FeF_3_ precipitates in the tribofilm.
